# Standardized high-throughput evaluation of cell-based compound screens

**DOI:** 10.1186/1471-2105-9-475

**Published:** 2008-11-12

**Authors:** Peter Frommolt, Roman K Thomas

**Affiliations:** 1 Institute of Medical Statistics, Informatics and Epidemiology, University of Köln, Köln, Germany; 2 Max Planck Institute of Neurological Research with Klaus Joachim Zülch laboratories of the Max Planck Society and the Medical Faculty of the University of Köln, Köln, Germany; 3 Department I of Internal Medicine and Center of Integrated Oncology, University of Köln, Köln, Germany; 4 Chemical Genomics Center of the Max Planck Society, Dortmund, Germany

## Abstract

**Background:**

High-throughput screening of pharmaceutical compound activity in tissue culture experiments requires time-consuming repeated analysis of the large amounts of data generated. Automation of the evaluation procedure and assessment of measurement accuracy can save time and improve the comparability of results.

**Results:**

We present a tool for simultaneous evaluation of an arbitrary number of compound screens including a standardized statistical validation. It is provided as a novel R package with a Tcl/Tk-based GUI for convenient use in the lab and runs on usual platforms like Linux, Windows and Mac OS. In a compound screen of lung cancer cells, the tool was successfully and efficiently applied for data analysis.

**Conclusion:**

The package provides an efficient and intuitive platform for automatic evaluation of compound screens, improving the performance and standardization of data analysis.

## Background

Cell-based screening of the cytotoxic activity of chemical compounds in cancer cells has emerged as a widely used method in the drug discovery process. Typically, cells are treated with several concentrations of compound in 96- or 384-well microtiter plates for a predefined time period. A common method to evaluate these experiments in a quantitative fashion is to determine a half-maximal inhibitory concentration (IC50) for which cell growth is inhibited by 50%. Comprehensive efforts have been focused on screening experiments with thousands of compounds in industrial laboratories as well as institutions of public health. A screen of 60 cancer cell lines with a large library of agents was supervised by the National Cancer Institute [1]. Yet, these compound screens lack a standardized tool and implementation for automatic high-throughput evaluation. We propose the methods and software applied for evaluation in a screen of non-small cell lung cancer (NSCLC) *in vitro *cell cultures as a standard for cell line screens in future. The implementation is available for download under the General Publice License (GPL).

## Implementation

### Evaluation and validation of compound screens

For *l *= 1, ..., *k*, consider the screen of the *l*th compound in log-transformed concentrations *X*_*lj *_with *j *= 1, ..., *m*_*l*_. On the other hand, denote by *Y*_*lij *_the observed proportion of cells still being alive under concentration *j *in the *i*th replicate where *i *= 1, ..., *n*_*l*_. This determines *n*_*l *_dose-response curves formed by the respective points (*X*_*l*1_, ..., *Y*_*li*1_), ..., (*X*_*lm*_, ..., *Y*_lim_). One IC50 value can be determined from each of these by the preimage *c*_*li *_of the 50% point under a linear spline. In real experiments, this value may not be uniquely determined as the curve crosses the 50% point several times. In these cases, it is most appropriate to define the IC50 value as the smallest concentration where this occurs. The resulting IC50 from the repeated screen is determined as the mean ${\bar{c}}_{l}$ of these *n*_*l *_concentrations with a 95% confidence interval

$$c_{l}\in \left[{\bar{c}}_{l}-1.96\frac{\hat{\sigma }}{\sqrt{n_{l}}},{\bar{c}}_{l}+1.96\frac{\hat{\sigma }}{\sqrt{n_{l}}}\right],$$

making use of the fact that the IC50 concentrations are normally distributed through the above logarithmic transformation which is inverted subsequently after analysis. Here, $\hat{\sigma }$ denotes the standard deviation of the *n*_*l *_values. If most samples are resistant towards a particular compound in the overall screen, we propose to determine the 25% inhibitory concentration (IC25 value) instead to get a more widespread profile for that sample. To guess the accuracy of an experiment, one point of interest is the variability of the resulting IC50 values. This can be determined by the coefficient of variation ${\hat{v}}_{l}$ of these. On the other hand, the standard deviations of the raw data ${\hat{\sigma }}_{lj}$ can be determined for each concentration to verify the initial validity of the measurements. As this results in a total of *m*_*l *_values, it is reasonable to regard the maximum ${\hat{\sigma }}_{l}:=\max\left\{{\hat{\sigma }}_{l1},...,{\hat{\sigma }}_{lm}\right\}$ of these values as the overall accuracy of the data points.

### Features of the R package

**Figure 1:**
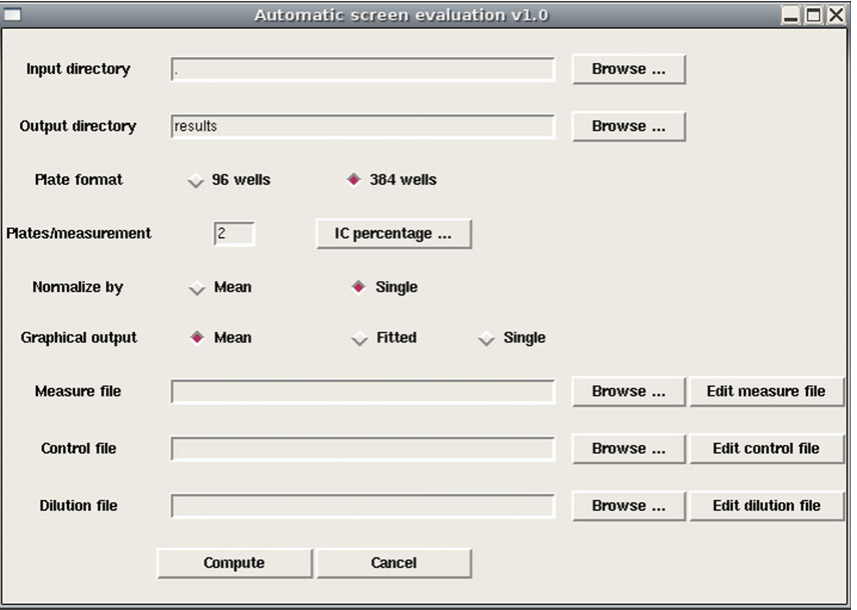
**Main window of the GUI-controlled package**. Screenshot of the main window of the GUI-controlled 'ic50' package. Features for specification of the experimental design are provided as well as options for evaluation. The use of the wells on the plates can be modified by specification of three previously created configuration files.

The novel add-on package 'ic50' is available for download from the Comprehensive R Archive Network (CRAN) and provides automatic performance of the above evaluation methods. The functions of the package are appropriate for immediate use on the R console but can be accessed by an intuitive GUI as well (Figure 1). The main feature that makes the described tool exceedingly useful for practice is that *all *data in an arbitrary directory on the local harddisk can be evaluated simultaneously by just one mouse click. In particular, the amount of data to be evaluated is not limited and may comprise screens of hundreds or thousands of compounds or samples, respectively, as long as the same design is shared by all experiments.

Microtiter plates with 96 or 384 wells are supported up to now. Raw data are expected to be passed as tab-delimited text files which are the typical output from appropriate microplate readers. The arrangement of the measurements on the well matrix can be different for each experimental setup. To address this, the design can be configured by three separate files, one specifying the coordinates of the wells for the actual compound measurements, one for the locations of control measurements to be used for normalization and a third for specification of the respective concentrations used for each measurement. Several samples of such files are distributed together with the package. Normalization with control wells can be performed by taking the mean of a specified control row or by one single control well per concentration, where wells can be used multiple times in both cases. Inhibitory percentages can be configured as 50% for all compounds, which is default, or any other individual value, e.g. to calculate IC25 values. Graphical output can be modified by additional options.

As for any R package, there is detailed documentation of all features available with additional examples for illustration and a step-by-step tutorial document guiding the user to prepare his data and configuration files for analysis with the tool (Additional file 1).

## Results and Discussion

**Figure 2:**
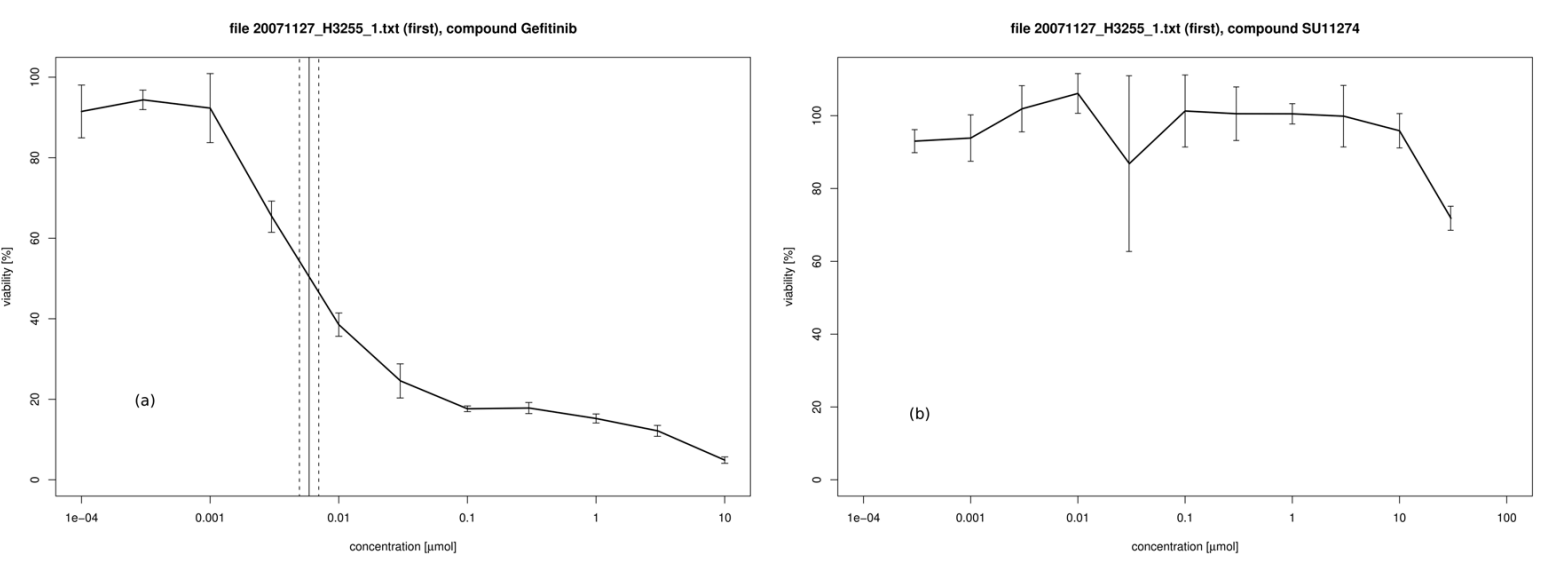
**Dose-response curves for H3255 cells under gefitinib and SU11274 treatment**. Graphical output for the H3255 NSCLC cell line treated **(a) **with gefitinib and **(b) **with SU11274. For gefitinib, the solid line denotes the IC50 value of the screen, whereas the 95% confidence bounds are given as dashed lines. These lines are not plotted for SU11274 as the sample is resistant to this treatment and there is no well-defined IC50 value. For each concentration, the standard deviation of the measurements is displayed as an error bar.

Results from an evaluation of the lung cancer cell line H3255 under treatment with 7 different compounds are given in Table 1 with the corresponding dose-response curves for gefitinib and SU11274 in Figure 2. The measurements were carried out using a Mithras LB 940 multimode reader (Berthold Technologies, Bad Wildbad, Germany) with the output files converted to tab-delimited text files before the procedure. In general, the numeric results are all given in one single text file with the structure of Table 1 and a graphics output as exemplified in Figure 2 is written to one single pdf file in the specified output directory for all compounds in the screen. The cell line H3255 carries an activating mutation of the EGFR gene making it sensitive to the EGFR inhibitors gefitinib and erlotinib [2]. The full data collection of this compound screen will be published elsewhere [3].

**Table 1:** Results for H3255 cells under gefitinib treatment

Compound	IC50	*c*_*low*_	*c*_*up*_		*v*
17AAG	0.0275	0.0237	0.0321	0.0901	0.0304
Gefitinib	0.0059	0.005	0.007	0.0858	0.0242
Purvalanol	5.689	3.850	8.406	0.1437	0.1618
Rapamycin	5.957	0.0267	1328	0.0815	2.183
SU11274	NA	NA	NA	0.2413	NA
UO126	8.414	7.218	9.808	0.1727	0.0518
VX680	2.692	0.9192	7.881	0.1226	0.7816

Results of the dose-response experiment for the EGFR-mutant H3255 NSCLC cell line treated with small molecule compounds 17AAG, purvalanol, SU11274, gefitinib, rapamycin, VX680 and U0126. The lowest IC50 value (first column) reveals strong activity of gefitinib, whereas the NA values indicate that the sample is resistant to SU11274 treatment and no IC50 value was calculated. The columns clow and cup show the 95% confidence interval for the IC50 values. The maximum of the standard deviations of the measurements is given in the column , whereas in the last column, v denotes the coefficient of variation of the IC50 values.

For a resistant sample, a typical curve looks like Figure 2b with no remarkable variation of viability over the concentrations. For the IC50 concentration, the tool returns a NA value in this case and does not include it into the plot. The same happens if the viability is almost constant at a somewhat lower percentage (Additional file 2, figure **(a) **and **(b)**). However, other kinds of unexpected behaviour may occur in real experiments. The curve can be non-monotonic and cross the 50% point several times (Additional file 2, figure **(c)**). As mentioned above, the smallest of the several concentrations is returned in this case. On the other hand, erroneous measurements may yield a monotonically increasing curve with viability below 50% even for small concentrations (Additional file 2, figure **(d)**). In this case, the tool returns a NA value for the IC50 concentration.

The lowest IC50 value in the H3255 cells was observed under gefitinib treatment, thus confirming the appropriateness of our screening and analytical approaches [2]. For the coefficient of variation, a usual standard is to require *v *< 0.05 for reasonable accuracy. Regarding the results in Table 1, the maximum standard deviation ranges between 0.0815 and 0.2413, suggesting an upper threshold of *τ *= 0.2 for validation. The measurements for rapamycin show very strong variability with an artificially wide confidence interval. For the cell line screen, this result was therefore discarded and replaced by a repeated experiment.

## Conclusion

In summary, the 'ic50' package provides a platform for time-efficient evaluation of cell-based compound screens. The experimental setup can be configured in any order and re-used for multiple subsequent analyses. A standardized validation is included in the tool and can be used to assess the accuracy of the experiments. The approach is suitable to confirm biological activity of targeted drugs in cancer cells with specific genetic lesions.

## Availability and requirements

The 'ic50' package is a platform-independent add-on to the R environment for statistical computing. It uses a Tcl/Tk-based GUI and is available at the URL  under the General Public License (GPL). There are no restrictions for its use. An installation of the R environment with Tcl/Tk support is required. The package is also available as additional material to this paper (Additional files 3 and 4).

## Authors' contributions

PF carried out the software programming and derived the statistical validation. RKT generated the lung cancer screen data and provided the platform for testing the appropriateness of the methods. The manuscript was written by both authors.

## Supplementary Material

Additional file 1 **Tutorial**. This document aims to help the user getting started with the package: the correct formatting of the input data and configuration files is explained in a step-by-step manual. Click here for file

Additional file 2 **Supplementary Figure**. This figure displays several situations with typical exceptions occuring in the measurements: **(a) **cell viability is essentially constant at 50%, **(b) **cell viability is essentially constant at 0%, **(c) **the curve crosses the 50% point several times, and **(d) **the percentage is monotonically increasing for increasing concentrations. Click here for file

Additional file 3 **Source code of the software**. The platform-independent source code for version 1.3 of the package is provided as a gzipped tar archive. Click here for file

Additional file 4 **Windows binary code of the software**. A pre-compiled version is provided for MS Windows. It can be installed from within the R environment on Windows systems. Click here for file

## References

[B1] Stinson SF, Alley MC, Kopp WC, Fiebig HH, Mullendore LA, Pittman AF, Kenney S, Keller J, Boyd MR (1992). Morphological and immunocytochemical characteristics of human tumor cell lines for use in a disease-oriented anticancer drug screen. Anticancer Res.

[B2] Sharma SV, Bell DW, Settleman J, Haber DA (2007). Epidermal growth factor receptor mutations in lung cancer. Nature Rev Cancer.

[B3] Michel K, Zander T, Frommolt P, Sos M, Weiß J, Mermel C, Koker M, Fischer S, Rauh D, Lin W, Winckler W, Shah K, LaFramboise T, Feng W, Hanna M, Tolosi L, Rahnenführer J, Verhaak R, Shimamura T, Beroukhim R, Chiang D, Getz G, Hellmich M, Wolf J, Girard L, Peyton M, Weir BA, Greulich H, Chen TH, Shapiro GI, Wong KK, Garraway L, Gazdar AF, Minna J, Thomas RK (2008). Predicting drug activity in non-small cell lung cancer based on genetic lesions.

[B4] Dalgaard P (2002). Introductory Statistics with R.

[B5] Gentleman SV, Carey JC, Bates D, Bolstad B, Dettling M, Dudoit S, Ellis B, Gautier L, Ge Y, Gentry J, Hornik K, Hothorn T, Huber W, Iacus S, Irizarry R, Leisch F, Li C, Maechler M, Rossini AJ, Sawitzki G, Smith C, Smyth G, Tierney L, Yang JYH, Zhang J (2004). Bioconductor: open software development for computational biology and bioinformatics. Genome Biol.

[B6] R Development Core Team (2008). R: A Language and Environment for Statistical Computing.

[B7] Tallarida RJ (2000). Drug synergism and dose-effect data analysis.

