# Supplementary material for: Standardized high-throughput evaluation of cell-based compound screens
Source: BMC Bioinformatics. 2008 Nov 12;9:475. doi: 10.1186/1471-2105-9-475 (PMC2639430; doi:10.1186/1471-2105-9-475)
Supplement: Additional file 1 — Tutorial. This document aims to help the user getting started with the package: the correct formatting of the input data and configuration files is explained in a step-by-step manual. [file 1471-2105-9-475-S1.pdf]

Analyzing data from a cell-based compound screen with the ic50 package: A tutorial

This document aims to help users of the `ic50` package to prepare their data and configuration of experimental setup for evaluation. The procedure is illustrated with data from a screen of non-small cell lung cancer (NSCLC) cell lines which are distributed together with the package. The data and configuration files are available in the `nsclc` and `design` folders. The GUI-guided procedure requires an installation of R with Tcl/Tk support on the local machine.
To get started, let's open the R environment and load the package by `library(ic50)`. The graphical user interface can be launched using the command `ic50()` which will show the main window (Figure 1). The several options will be explained in the following, along with detailed information on how the data and configuration have to be prepared.

1 Measurement data accepted as input

The package expects raw data from a 384- or 96-well microplate reader used for measuring cell viability in the respective wells by light intensity. For each of the 384 or 96 wells, there is one intensity value measured. The data are expected as an ASCII/Unicode textfile containing a 16x24 matrix (384 wells) or a 8x12 matrix (96 wells) of numeric intensities with tabulators between the fields for the respective wells. The lung cancer screen was evaluated using a Mithras LB 940 (Berthold Technologies, Bad Wildbad, Germany) multimode reader. This device produces Microsoft Excel spreadsheets as output which must be converted to a text-only format as support for Excel sheets in R is rather limited. For the A549 cancer cell line, the resulting file looks like

```
770190  777340  687320  740850  668800   723760  792550   763770  735900  725320  719830   ...
729850  720250  777740  711370  733620   756260  762100   767090  776420  755660  741920   ...
769350  784600  784630  705410  1012810  720140  886930   848170  722690  852540  1079420  ...
797420  710240  796120  796100  740330   671130  838680   752940  797670  778580  811000   ...
773180  778890  748930  853250  741580   820780  738490   867720  776020  803810  792130   ...
832550  767920  726140  945310  795530   926020  760650   869280  775120  932400  886850   ...
826170  814910  834150  751960  881390   809410  826010   820340  833960  850080  787560   ...
814500  818000  835790  769010  828060   803220  774790   837590  856000  819810  788120   ...
703510  840540  824430  878490  819340   773210  1015190  863210  778320  858040  791310   ...
851970  791030  730540  780720  762970   997440  815560   840010  839880  800010  749650   ...
798510  745970  626340  807560  762300   716520  1159410  921760  766570  697820  1114720  ...
766310  693300  726160  748910  703550   767480  681780   738830  765700  768680  705880   ...
774330  679550  736380  897880  759880   857710  694580   838290  695910  847250  715010   ...
723000  754910  704430  770170  729360   724990  708800   733380  687940  801490  734150   ...
712590  786800  760180  715530  671120   798410  794700   769600  752520  816930  772930   ...
867310  688120  812220  700140  792560   768690  848370   793570  784700  746180  747950   ...,
```

where only the first 11 of 24 columns are shown. The user should format his data according to the above requirements. If several plates are used to evaluate replicate measurements, their filenames must be next to another in alphabetical order to enable automatic identification of plates belonging together. As a reference, example files are

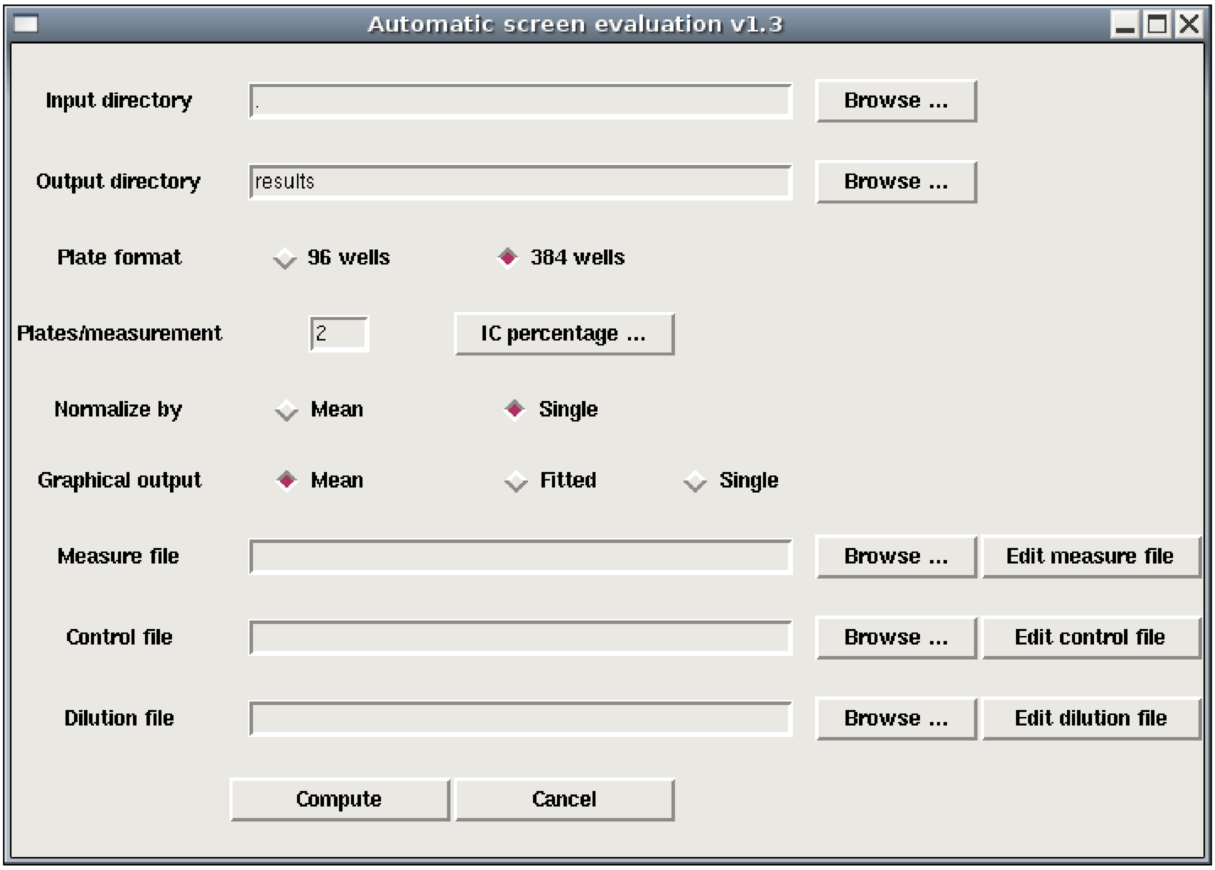

Figure 1: Main window of the IC50 tool with fields for choosing the input and configuration files and options for the microplate layout and graphical output.

available in the subfolder `nsclc` of the package. To get started with the evaluation procedure, we create a directory `rawdata` on the harddisk and copy all the raw data into this. For the example, we use the 8 files from the `nsclc` folder.

2 Preparation of the configuration files

There are three text files to be modified according to the plate layout. The *measure* file gives information on the locations of the viability measurements on the plate. The *control* file configures the wells being used for normalization. Finally, the concentrations used for each series are specified in the *dilution* file. These can either be created in a text editor of the user's choice or opened during the interactive process by clicking the *Edit measure file* etc. buttons. The latter will open a window typically used to edit matrices and data sets in R (Figure 2) in which the configuration can be edited according to the explanations given below. Subsequently, the window is closed by clicking the *Quit*

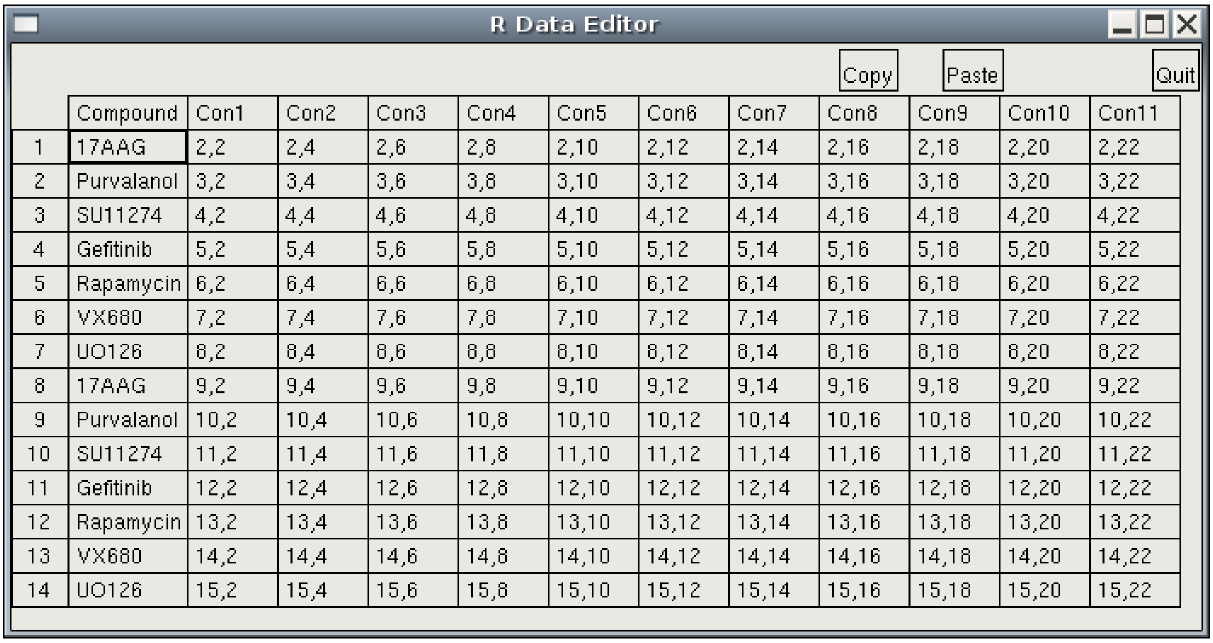

	Compound	Con1	Con2	Con3	Con4	Con5	Con6	Con7	Con8	Con9	Con10	Con11
1	17AAG	2,2	2,4	2,6	2,8	2,10	2,12	2,14	2,16	2,18	2,20	2,22
2	Purvalanol	3,2	3,4	3,6	3,8	3,10	3,12	3,14	3,16	3,18	3,20	3,22
3	SU11274	4,2	4,4	4,6	4,8	4,10	4,12	4,14	4,16	4,18	4,20	4,22
4	Gefitinib	5,2	5,4	5,6	5,8	5,10	5,12	5,14	5,16	5,18	5,20	5,22
5	Rapamycin	6,2	6,4	6,6	6,8	6,10	6,12	6,14	6,16	6,18	6,20	6,22
6	VX680	7,2	7,4	7,6	7,8	7,10	7,12	7,14	7,16	7,18	7,20	7,22
7	UO126	8,2	8,4	8,6	8,8	8,10	8,12	8,14	8,16	8,18	8,20	8,22
8	17AAG	9,2	9,4	9,6	9,8	9,10	9,12	9,14	9,16	9,18	9,20	9,22
9	Purvalanol	10,2	10,4	10,6	10,8	10,10	10,12	10,14	10,16	10,18	10,20	10,22
10	SU11274	11,2	11,4	11,6	11,8	11,10	11,12	11,14	11,16	11,18	11,20	11,22
11	Gefitinib	12,2	12,4	12,6	12,8	12,10	12,12	12,14	12,16	12,18	12,20	12,22
12	Rapamycin	13,2	13,4	13,6	13,8	13,10	13,12	13,14	13,16	13,18	13,20	13,22
13	VX680	14,2	14,4	14,6	14,8	14,10	14,12	14,14	14,16	14,18	14,20	14,22
14	UO126	15,2	15,4	15,6	15,8	15,10	15,12	15,14	15,16	15,18	15,20	15,22

Figure 2: R Data Editor window for modification of the configuration files from within the interactive process of the IC50 package.

button. Microsoft Excel or Word are not recommended for modification of the files as well as the corresponding OpenOffice programs because the position of tabulators and newline signs cannot sufficiently controlled by the user. Instead, a simple text editor like gedit or more sophisticated programs like emacs or WinEdt should be used.
Any row in the configuration files starts with the name of the compound to which the row refers, between quotes, and a tabulator separating the name from the row itself. For the three files, we create a new folder `config`.

2.1 *Measure* file

For each measurement series, the wells being used are specified in a corresponding row of the *measure* file. The compound name is given between quotes, followed by a list of comma-delimited pairs of numbers which specify the coordinates of the wells. In the lung cancer example, 11 concentrations of 17AAG were screened in the wells with coordinates (2,2), (2,4), (2,6), (2,8), (2,10), (2,12), (2,14), (2,16), (2,18), (2,20) and (2,22). Thus the first row of the measure file is the tab-delimited list

```
"17AAG"   2,2   2,4   2,6   2,8   2,10   2,12   2,14   2,16   2,18   2,20   2,22
```

The line must be finished after the last statement without a tabulator behind it. Now, there were another 6 compounds screened one below the other on this plate with 2 repli-

cates each. As the arrangement is the same for these but a new row of the plate is used for each series, the coordinates for the next compounds start with (3,2), (4,2), (5,2), ..., and the complete file looks like

```
"17AAG"        2,2   2,4   2,6   2,8   2,10   2,12   2,14   2,16   2,18   2,20   2,22
"Purvalanol"   3,2   3,4   3,6   3,8   3,10   3,12   3,14   3,16   3,18   3,20   3,22
"SU11274"      4,2   4,4   4,6   4,8   4,10   4,12   4,14   4,16   4,18   4,20   4,22
"Gefitinib"    5,2   5,4   5,6   5,8   5,10   5,12   5,14   5,16   5,18   5,20   5,22
"Rapamycin"    6,2   6,4   6,6   6,8   6,10   6,12   6,14   6,16   6,18   6,20   6,22
"VX680"        7,2   7,4   7,6   7,8   7,10   7,12   7,14   7,16   7,18   7,20   7,22
"U0126"        8,2   8,4   8,6   8,8   8,10   8,12   8,14   8,16   8,18   8,20   8,22
"17AAG"        9,2   9,4   9,6   9,8   9,10   9,12   9,14   9,16   9,18   9,20   9,22
"Purvalanol"   10,2  10,4  10,6  10,8  10,10  10,12  10,14  10,16  10,18  10,20  10,22
"SU11274"      11,2  11,4  11,6  11,8  11,10  11,12  11,14  11,16  11,18  11,20  11,22
"Gefitinib"    12,2  12,4  12,6  12,8  12,10  12,12  12,14  12,16  12,18  12,20  12,22
"Rapamycin"    13,2  13,4  13,6  13,8  13,10  13,12  13,14  13,16  13,18  13,20  13,22
"VX680"        14,2  14,4  14,6  14,8  14,10  14,12  14,14  14,16  14,18  14,20  14,22
"U0126"        15,2  15,4  15,6  15,8  15,10  15,12  15,14  15,16  15,18  15,20  15,22
```

We save this under the filename `measure_example.txt` in the `config` folder. If one compound is applied several times, the package automatically considers these rows as replicates of the same compound. In the example, four replicates of each compound were measured and distributed over two raw data files with the above layout, i.e. with two replicates in each file.
To create the configuration for his own data, the user must include one row for each measurement series, starting with the compound name between quotes followed by a tab-delimited list of the well coordinates used for the respective concentrations. Importantly, the spelling of the name in two rows representing the same compound must be exactly equal, including blanks or upper and lower case, and each row must be finished after the last statement without a tabulator.

2.2 *Control* file

As the intensity itself is not informative for the cell viability, any measured concentration needs a reference for normalization. In the lung cancer screen, control cells were treated by 0.1% Dimethylsulfoxyd (DMSO) dilution and one control well was used for each measurement well: the 11 measurements under 17AAG treatment must be divided by the values in the 11 DMSO-treated wells (2,3), (2,5), (2,7), (2,9), (2,11), (2,13), (2,15), (2,17), (2,19), (2,21) and (2,23). The remaining series, including the replicates, have their own normalization wells. Taken together, the control file for the lung cancer screen looks like

```
"17AAG"        2,3    2,5    2,7    2,9    2,11    2,13    2,15    2,17    2,19    2,21    2,23
"Purvalanol"   3,3    3,5    3,7    3,9    3,11    3,13    3,15    3,17    3,19    3,21    3,23
"SU11274"      4,3    4,5    4,7    4,9    4,11    4,13    4,15    4,17    4,19    4,21    4,23
"Gefitinib"    5,3    5,5    5,7    5,9    5,11    5,13    5,15    5,17    5,19    5,21    5,23
"Rapamycin"    6,3    6,5    6,7    6,9    6,11    6,13    6,15    6,17    6,19    6,21    6,23
"VX680"        7,3    7,5    7,7    7,9    7,11    7,13    7,15    7,17    7,19    7,21    7,23
"U0126"        8,3    8,5    8,7    8,9    8,11    8,13    8,15    8,17    8,19    8,21    8,23
"17AAG"        9,3    9,5    9,7    9,9    9,11    9,13    9,15    9,17    9,19    9,21    9,23
"Purvalanol"   10,3   10,5   10,7   10,9   10,11   10,13   10,15   10,17   10,19   10,21   10,23
"SU11274"      11,3   11,5   11,7   11,9   11,11   11,13   11,15   11,17   11,19   11,21   11,23
"Gefitinib"    12,3   12,5   12,7   12,9   12,11   12,13   12,15   12,17   12,19   12,21   12,23
"Rapamycin"    13,3   13,5   13,7   13,9   13,11   13,13   13,15   13,17   13,19   13,21   13,23
"VX680"        14,3   14,5   14,7   14,9   14,11   14,13   14,15   14,17   14,19   14,21   14,23
"U0126"        15,3   15,5   15,7   15,9   15,11   15,13   15,15   15,17   15,19   15,21   15,23
```

We save this in the `design` folder using the file name `control_example.txt`. For this layout, the option *Normalize by Single* must be chosen in the GUI which refers to the fact that each well measured has got its own normalization well. Likewise, the experimenter may wish to have only a limited number of wells used for normalization. In this case, the coordinates of these can be specified analogously and used multiple times: the *control* file

```
"17AAG"        2,3   2,5   2,7   2,9   2,11   2,13   2,15   2,17   2,19   2,21   2,23
"Purvalanol"   2,3   2,5   2,7   2,9   2,11   2,13   2,15   2,17   2,19   2,21   2,23
"SU11274"      2,3   2,5   2,7   2,9   2,11   2,13   2,15   2,17   2,19   2,21   2,23
"Gefitinib"    2,3   2,5   2,7   2,9   2,11   2,13   2,15   2,17   2,19   2,21   2,23
"Rapamycin"    2,3   2,5   2,7   2,9   2,11   2,13   2,15   2,17   2,19   2,21   2,23
"VX680"        2,3   2,5   2,7   2,9   2,11   2,13   2,15   2,17   2,19   2,21   2,23
"U0126"        2,3   2,5   2,7   2,9   2,11   2,13   2,15   2,17   2,19   2,21   2,23
"17AAG"        2,3   2,5   2,7   2,9   2,11   2,13   2,15   2,17   2,19   2,21   2,23
"Purvalanol"   2,3   2,5   2,7   2,9   2,11   2,13   2,15   2,17   2,19   2,21   2,23
"SU11274"      2,3   2,5   2,7   2,9   2,11   2,13   2,15   2,17   2,19   2,21   2,23
"Gefitinib"    2,3   2,5   2,7   2,9   2,11   2,13   2,15   2,17   2,19   2,21   2,23
"Rapamycin"    2,3   2,5   2,7   2,9   2,11   2,13   2,15   2,17   2,19   2,21   2,23
"VX680"        2,3   2,5   2,7   2,9   2,11   2,13   2,15   2,17   2,19   2,21   2,23
"U0126"        2,3   2,5   2,7   2,9   2,11   2,13   2,15   2,17   2,19   2,21   2,23
```

will use the wells (2,3), (2,5), (2,7), (2,9), (2,11), (2,13), (2,15), (2,17), (2,19), (2,21) and (2,23) for normalization of all measurements. If, on the other hand, there are less control than measurement wells, the option *Normalize by Mean* can be used. In this approach, the mean value of the specified control wells is used for normalization. For example, the *control* file

```
"17AAG"        2,3    2,5    2,7    2,9
"Purvalanol"   3,3    3,5    3,7    3,9
"SU11274"      4,3    4,5    4,7    4,9
"Gefitinib"    5,3    5,5    5,7    5,9
"Rapamycin"    6,3    6,5    6,7    6,9
"VX680"        7,3    7,5    7,7    7,9
"U0126"        8,3    8,5    8,7    8,9
"17AAG"        9,3    9,5    9,7    9,9
"Purvalanol"   10,3   10,5   10,7   10,9
"SU11274"      11,3   11,5   11,7   11,9
"Gefitinib"    12,3   12,5   12,7   12,9
"Rapamycin"    13,3   13,5   13,7   13,9
"VX680"        14,3   14,5   14,7   14,9
"U0126"        15,3   15,5   15,7   15,9
```

uses the mean of the wells (2,3), (2,5), (2,7) and (2,9) for normalization of the 17AAG

measurements (first replicate), the mean of (3,3), (3,5), (3,7) and (3,9) for Purvalanol (first replicate) etc. if *Normalize by Mean* is chosen. Otherwise, the evaluation will terminate with an error message.
If for each measurement series, the user has as many control wells as measurements, the normalization should be based on the *Normalize by Single* approach. The number of wells must then be the same in each row of the *measure* and *control* files. If the *Normalize by Mean* approach is chosen, this is not necessary and the number of wells for normalization is arbitrary. In both cases it is, however, crucial that the spelling of the compound names is exactly the same in both files as otherwise the software cannot identify the rows belonging together. No additional misplaced tabulators or blanks should be in the file.

2.3 *Dilution* file

The seven compounds in the lung cancer screen were applied in maximum doses of 10 μmol (17AAG, gefitinib, rapamycin), 30 μmol (SU11274), 60 μmol (VX680) or 90 μmol (Purvalanol), respectively. For the remaining wells, concentrations were decreased by factor three, e.g. for 17AAG, the dilution series 0.0001, 0.0003, 0.001, 0.003, 0.1, 0.3, 1, 3 and 10 μmol was used. For each compound, the corresponding row is now specified as a tab-delimited series in the *dilution* file, starting with the compound name itself between quotes and a tabulator:

```
"17AAG"        0.0001  0.0003  0.001   0.003   0.01   0.03   0.1   0.3   1    3    10
"Purvalanol"   0.001   0.003   0.01    0.03    0.1    0.3    1     3     10   30   90
"SU11274"      0.0003  0.001   0.003   0.01    0.03   0.1    0.3   1     3    10   30
"Gefitinib"    0.0001  0.0003  0.001   0.003   0.01   0.03   0.1   0.3   1    3    10
"Rapamycin"    0.0001  0.0003  0.001   0.003   0.01   0.03   0.1   0.3   1    3    10
"VX680"        0.0006  0.002   0.006   0.02    0.06   0.2    0.6   2     6    20   60
"UO126"        0.001   0.003   0.01    0.03    0.1    0.3    1     3     10   30   90
"17AAG"        0.0001  0.0003  0.001   0.003   0.01   0.03   0.1   0.3   1    3    10
"Purvalanol"   0.001   0.003   0.01    0.03    0.1    0.3    1     3     10   30   90
"SU11274"      0.0003  0.001   0.003   0.01    0.03   0.1    0.3   1     3    10   30
"Gefitinib"    0.0001  0.0003  0.001   0.003   0.01   0.03   0.1   0.3   1    3    10
"Rapamycin"    0.0001  0.0003  0.001   0.003   0.01   0.03   0.1   0.3   1    3    10
"VX680"        0.0006  0.002   0.006   0.02    0.06   0.2    0.6   2     6    20   60
"UO126"        0.001   0.003   0.01    0.03    0.1    0.3    1     3     10   30   90
```

We save this as a text file `dilution_example.txt` to the folder `config`. When creating this file for his own screen, the user should bear in mind that each series specified in the *measure* file needs its own row in the *dilution* file with the same number of concentrations as wells in the *measure* file. To enable the software to link the corresponding rows in the *measure*, *control* and *dilution* files, it is crucial that the spelling of the compound names is exactly the same for multiple replicates as well as between the three files.

3 High-throughput evaluation and validation

After having prepared the input data and configuration files, there are just a few mouse clicks left to finish the evaluation. We open the GUI by the command `ic50()` (Figure 1).

For the lung cancer example,

- we select the folder `rawdata` as the input directory,
- leave the output directory as it is,
- choose 16x24, i.e. 384 wells, as the plate format for the data,
- perform the evaluation for pairs of two plates each as four replicates were measured with two on each plate,
- leave the *IC percentage* setting as it is because we wish to calculate the IC50 concentrations. The button can be used to choose a different inhibitory percentage, e.g. if the IC25 concentration must be evaluated. Next,
- we choose *Single* as the normalization method as explained above,
- the *Graphical output* option allows to display either a curve of the mean IC50 values with error bars over the replicates, a curve with a logistic model fitting or a plot of the single curves from the respective replicates and finally,
- we select the three files `measure_example.txt`, `control_example.txt` and `dilution_example.txt` for the configuration.

Clicking the *Compute* button starts the evaluation. The results are written to the console as well as the selected output folder. A table with columns representing the IC50 concentrations, 95% confidence limits for these, the maximum of the standard deviations for the data points and the coefficient of variation of the replicate IC50 values is given as well as the dose-response curves in a pdf document.

Now, once an appropriate experimental layout has been found, this is typically not changed for further compound screens. The configuration files can therefore just be left as they are with only the compound names being changed everywhere. The most recently used files are saved in the current workspace directory as the hidden files `.last384_measure.txt`, `.last384_control.txt` and `.last384_dilution.txt`.

It should be mentioned that the graphical user interface is in fact nothing more than a front-end to the R functions `hts.384` and `hts.96` which apply to the same problem and do the same evaluation. The use of these functions is extensively described in the manual pages of the package.
