# Supplementary figures and images for: Standardized high-throughput evaluation of cell-based compound screens
Source: BMC Bioinformatics. 2008 Nov 12;9:475. doi: 10.1186/1471-2105-9-475 (PMC2639430; doi:10.1186/1471-2105-9-475)

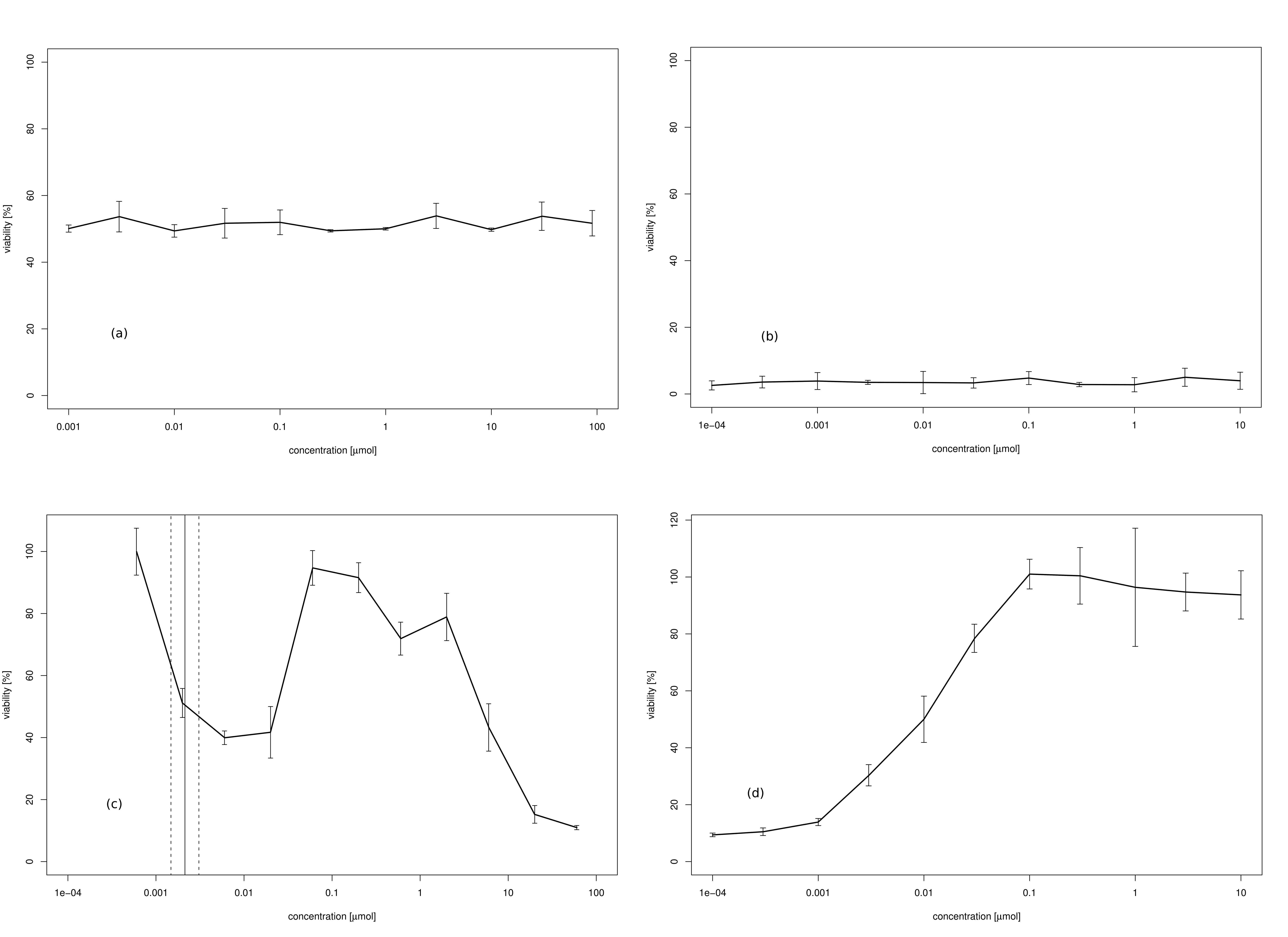

Supplement: Additional file 2 — Supplementary Figure. This figure displays several situations with typical exceptions occuring in the measurements: (a) cell viability is essentially constant at 50%, (b) cell viability is essentially constant at 0%, (c) the curve crosses the 50% point several times, and (d) the percentage is monotonically increasing for increasing concentrations. [file 1471-2105-9-475-S2.jpeg]
