# Supplementary material for: Standardized high-throughput evaluation of cell-based compound screens
Source: BMC Bioinformatics. 2008 Nov 12;9:475. doi: 10.1186/1471-2105-9-475 (PMC2639430; doi:10.1186/1471-2105-9-475)
Supplement: Additional file 4 — Windows binary code of the software. A pre-compiled version is provided for MS Windows. It can be installed from within the R environment on Windows systems. [file 1471-2105-9-475-S4.zip › ic50/doc/index.html]

R: ic50 vignettes

## Vignettes of package ic50

Sorry, the package contains no vignette meta-information or index.
Please browse the directory.
