# Supplementary material for: Standardized high-throughput evaluation of cell-based compound screens
Source: BMC Bioinformatics. 2008 Nov 12;9:475. doi: 10.1186/1471-2105-9-475 (PMC2639430; doi:10.1186/1471-2105-9-475)
Supplement: Additional file 4 — Windows binary code of the software. A pre-compiled version is provided for MS Windows. It can be installed from within the R environment on Windows systems. [file 1471-2105-9-475-S4.zip › ic50/html/00Index.html]

R: Standardized high-throughput evaluation of compound screens

# Standardized high-throughput evaluation of compound screens

---

## Documentation for package ‘ic50’ version 1.3

## User Guides and Package Vignettes

Read overview or browse directory.

## Help Pages

|  |  |
| --- | --- |
| A549\_1 | Results from a compound screen on 84 NSCLC cell lines. |
| A549\_2 | Results from a compound screen on 84 NSCLC cell lines. |
| Calu1\_1 | Results from a compound screen on 84 NSCLC cell lines. |
| Calu1\_2 | Results from a compound screen on 84 NSCLC cell lines. |
| default384\_control | Configuration files specifying the design of a compound screen on 84 NSCLC cell lines |
| default384\_dilution | Configuration files specifying the design of a compound screen on 84 NSCLC cell lines |
| default384\_measure | Configuration files specifying the design of a compound screen on 84 NSCLC cell lines |
| default96\_control | Configuration files specifying the design of a compound screen on 84 NSCLC cell lines |
| default96\_dilution | Configuration files specifying the design of a compound screen on 84 NSCLC cell lines |
| default96\_measure | Configuration files specifying the design of a compound screen on 84 NSCLC cell lines |
| H322\_1 | Results from a compound screen on 84 NSCLC cell lines. |
| H322\_2 | Results from a compound screen on 84 NSCLC cell lines. |
| HCC2429\_1 | Results from a compound screen on 84 NSCLC cell lines. |
| HCC2429\_2 | Results from a compound screen on 84 NSCLC cell lines. |
| hts.384 | Standardized high-throughput evaluation of compound screens |
| hts.96 | Standardized high-throughput evaluation of compound screens |
| ic50 | Standardized high-throughput evaluation of compound screens |
| ic50.384 | Standardized evaluation of compound screens |
| ic50.96 | Standardized evaluation of compound screens |
| mpi384\_control | Configuration files specifying the design of a compound screen on 84 NSCLC cell lines |
| mpi384\_dilution | Configuration files specifying the design of a compound screen on 84 NSCLC cell lines |
| mpi384\_measure | Configuration files specifying the design of a compound screen on 84 NSCLC cell lines |
| mpi96\_control | Configuration files specifying the design of a compound screen on 84 NSCLC cell lines |
| mpi96\_dilution | Configuration files specifying the design of a compound screen on 84 NSCLC cell lines |
| mpi96\_measure | Configuration files specifying the design of a compound screen on 84 NSCLC cell lines |
