# Supplementary material for: Standardized high-throughput evaluation of cell-based compound screens
Source: BMC Bioinformatics. 2008 Nov 12;9:475. doi: 10.1186/1471-2105-9-475 (PMC2639430; doi:10.1186/1471-2105-9-475)
Supplement: Additional file 4 — Windows binary code of the software. A pre-compiled version is provided for MS Windows. It can be installed from within the R environment on Windows systems. [file 1471-2105-9-475-S4.zip › ic50/html/design.html]

R: Configuration files specifying the design of a compound screen on
84 NSCLC cell lines

|  |  |
| --- | --- |
| design {ic50} | R Documentation |

## Configuration files specifying the design of a compound screen on 84 NSCLC cell lines

### Description

The experimental setup in a compound screen on 84 non-small cell lung
cancer (NSCLC) cell lines was designed according to the arrangement
specified in these data sets. However, the functions for screen
evaluation actually expect this information as files on the local
harddisk such as `"mpi384_measure.txt"`, `"mpi384_control.txt"` and
`"mpi384_dilution.txt"` which are installed together with the
package. Default examples of these configuration files are included as
`"default96_measure.txt"`, `"default384_measure.txt"` etc.

In the `"measure"` file, there must be one row specified for each
measurement series. This consists of the respective compound name
followed by tab-delimited information on the wells where the
measurements are located. Each of these must be given as a comma-delimited
pair of coordinates. The same is expected for the control wells
in the `"control"` file. Here, a particular well can be specified
multiple times and will then be repeatedly used as a control well for
the signal intensity without a compound being applied. If the
`normalize` argument to the functions is specified as `"single"`, there must be
one control well for each single measurement; if on the other hand,
`"mean"` is selected, an arbitrary number of wells can be
specified and the mean of those values is used for
normalization. Finally, the number of rows and the row names in the
`"dilution"` file must equal those in the preceding two
configuration files. Each row contains the compound name followed by a
tab-delimited list of the concentrations used for the respective
measurement series. It should by obvious that the number of concentrations in one row must equal the
number of wells in the `"measure"` file for each row. However,
the number of control wells can be distinct from these if the
`normalize` argument is set to `"mean"` such that the mean
of the respective control row is taken.

Importantly, the number of rows must be equal in all three files as
well as the row names, where case-sensitivity and literal equality has
to be carefully verified. The easiest way to create the configuration
files is to simply start the GUI using `ic50()` which
automatically creates a default version to be modified by the
user. After having saved this configuration, it can be repeatedly used
for screen evaluations as long as the experimental setup is not
changed.

A step-by-step tutorial document describing how to prepare the data and
configuration is included in the `ic50` package.

---

[Package *ic50* version 1.3 Index]
