# Supplementary material for: Standardized high-throughput evaluation of cell-based compound screens
Source: BMC Bioinformatics. 2008 Nov 12;9:475. doi: 10.1186/1471-2105-9-475 (PMC2639430; doi:10.1186/1471-2105-9-475)
Supplement: Additional file 4 — Windows binary code of the software. A pre-compiled version is provided for MS Windows. It can be installed from within the R environment on Windows systems. [file 1471-2105-9-475-S4.zip › ic50/html/hts.html]

R: Standardized high-throughput evaluation of compound screens

|  |  |
| --- | --- |
| hts {ic50} | R Documentation |

## Standardized high-throughput evaluation of compound screens

### Description

Simultaneous evaluation of a large number of compound screens on 96- and 384-well plates.

### Usage

```
ic50()
hts.96(indir=".",plates=2,measure=NULL,control=NULL,dilution=NULL,inhib=NULL,
       normalize="mean",graphics="mean",outdir="./results")
hts.384(indir=".",plates=2,measure=NULL,control=NULL,dilution=NULL,inhib=NULL,
        normalize="single",graphics="mean",outdir="./results")
```

### Arguments

|  |  |
| --- | --- |
| `indir` | A character specifying the directory which contains the raw data files. |
| `plates` | Number of plates used for each experiment. |
| `measure` | Configuration file for the locations of the measurement wells. |
| `control` | Configuration file for the locations of the control wells. |
| `dilution` | Configuration for the concentrations in each measurement. |
| `inhib` | Vector of real numbers between 0 and 1 specifying the percentage of inhibition to compute concentrations for. Defaults to 0.5 for all compounds. |
| `normalize` | Method to normalize the measurement by the controls. If `"mean"`, the mean of the controls specified by `control` is used; `"single"` requires one individual control well per measurement well. |
| `graphics` | A character specifying the plotting method. For `"mean"`, a dose-response curve of the mean values of the measurement series is given, whereas one curve is plotted for each if `"single"` is specified. For `"fitted"`, a sigmoid-shaped derivation of the logistic model is fitted to the data. |
| `outdir` | The directory where the results will be written. |

### Details

In cytotoxicity screens of chemical compounds, biological activity is
typically quantified by the concentration for which a particular
fraction (typically 0.5) of cell growth is inhibited after a predefined
treatment period. For this purpose, all concentrations are plotted
against the percentages of cells still being alive under this
treatment, forming a dose-response curve under which the preimage of the 0.5
point is defined as the half-maximum inhibitory concentration
(IC50). For high-throughput screens (HTS), in particular, the
evaluation of the data needs to be performed in an automatic fashion.

The `hts.96` and `hts.384` functions provide a powerful tool
to simultaneously evaluate all data in the specified input directory
`indir`. The data files are handled in groups of the size specified
by `plates` and the file names should be arranged in a way that
two plates with replicates for the same measurements
are displayed one below the other in a file browser. The data are
expected to be arranged in tab-delimited text files which is the typical
output of appropriate microplate readers. Just as for the
evaluation of a single measurement, the design must be specified by
tab-delimited files for `measure`, `control` and
`dilution`. Details on these are given in the manual of the
`default384_measure` and `default384_control`
files. In addition, a tutorial document
describing how to prepare the data and configuration is included in
the `ic50` package.

For each compound in the screen and each group of data files, a
graphics output is given in the file `"dose_response_curves.pdf"` in the
current workspace directory. In addition, the text file
`"ic50.txt"` contains a tab-delimted table with the same
evaluation as for the `ic50.96` and `ic50.384`
functions but for all experiments one below the other.

`ic50()` starts a GUI-based version of the `hts.96` and
`hts.384` functions. Preliminary change of the workspace
directory to the folder containing the data will remarkably reduce the
number of mouse clicks.

### Value

A data frame with the following columns:

|  |  |
| --- | --- |
| `first_file` | Filename of the respective first input file. |
| `compound` | Compound names. |
| `ic50` | The inhibitory concentrations for the respective compounds. |
| `clow` | Lower 0.95 confidence limits for the IC values. |
| `cup` | Upper 0.95 confidence limits for the IC values. |
| `maxsd` | Maximum of the standard deviations at the measured concentrations as determined from the single replicates. |
| `cv` | Coefficient of variation of the IC values as determined from the single replicates. |

### Author(s)

Peter Frommolt, University of Cologne peter.frommolt@uni-koeln.de  
http://www.medizin.uni-koeln.de/kai/imsie/homepages/Peter.Frommolt/

### Examples

```
#Example from a non-small cell lung cancer (NSCLC) cell line screen. In
#total, 84 samples were screened. The evaluation is exemplarily shown for
#the cell lines A549, Calu1, H322 and HCC2429.

data(A549_1,A549_2,Calu1_1,Calu1_2,H322_1,H322_2,HCC2429_1,HCC2429_2)
dir.create("NSCLC_screen")
write.table(A549_1,file="NSCLC_screen/A549_1.txt",row.names=FALSE,col.names=FALSE,sep="\t")
write.table(A549_2,file="NSCLC_screen/A549_2.txt",row.names=FALSE,col.names=FALSE,sep="\t")
write.table(Calu1_1,file="NSCLC_screen/Calu1_1.txt",row.names=FALSE,col.names=FALSE,sep="\t")
write.table(Calu1_2,file="NSCLC_screen/Calu1_2.txt",row.names=FALSE,col.names=FALSE,sep="\t")
write.table(H322_1,file="NSCLC_screen/H322_1.txt",row.names=FALSE,col.names=FALSE,sep="\t")
write.table(H322_2,file="NSCLC_screen/H322_2.txt",row.names=FALSE,col.names=FALSE,sep="\t")
write.table(HCC2429_1,file="NSCLC_screen/HCC2429_1.txt",row.names=FALSE,col.names=FALSE,sep="\t")
write.table(HCC2429_2,file="NSCLC_screen/HCC2429_2.txt",row.names=FALSE,col.names=FALSE,sep="\t")

data(mpi384_measure,mpi384_control,mpi384_dilution)
write.table(mpi384_measure,file="mpi384_measure.txt",row.names=FALSE,col.names=FALSE,sep="\t")
write.table(mpi384_control,file="mpi384_control.txt",row.names=FALSE,col.names=FALSE,sep="\t")
write.table(mpi384_dilution,file="mpi384_dilution.txt",row.names=FALSE,col.names=FALSE,sep="\t")

print(hts.384(indir="NSCLC_screen",
              measure="mpi384_measure.txt",control="mpi384_control.txt",dilution="mpi384_dilution.txt",
              inhib=rep(0.5,7),outdir="NSCLC_results",normalize="mean"))
```

---

[Package *ic50* version 1.3 Index]
