# Supplementary material for: Standardized high-throughput evaluation of cell-based compound screens
Source: BMC Bioinformatics. 2008 Nov 12;9:475. doi: 10.1186/1471-2105-9-475 (PMC2639430; doi:10.1186/1471-2105-9-475)
Supplement: Additional file 4 — Windows binary code of the software. A pre-compiled version is provided for MS Windows. It can be installed from within the R environment on Windows systems. [file 1471-2105-9-475-S4.zip › ic50/html/ic50.html]

R: Standardized evaluation of compound screens

|  |  |
| --- | --- |
| ic50 {ic50} | R Documentation |

## Standardized evaluation of compound screens

### Description

Calculation of IC50 values, automatic drawing of dose-response curves and
validation of compound screens on 96- and 384-well plates.

### Usage

```
ic50.96(files,measure=NULL,control=NULL,dilution=NULL,inhib=NULL,
        normalize="mean",graphics="mean",outdir="./results")
ic50.384(files,measure=NULL,control=NULL,dilution=NULL,inhib=NULL,
         normalize="single",graphics="mean",outdir="./results")
```

### Arguments

|  |  |
| --- | --- |
| `files` | Character vector of files containing the raw data. |
| `measure` | Configuration file for the locations of the measurement wells. |
| `control` | Configuration file for the locations of the control wells. |
| `dilution` | Configuration file for the concentrations in each measurement. See details below. |
| `inhib` | Vector of real numbers between 0 and 1 specifying the percentage of inhibition to compute concentrations for. Defaults to 0.5 for all compounds. |
| `normalize` | Method to normalize the measurement by the controls. For `"mean"`, the mean of the controls specified by `control` is used; `"single"` requires one individual control well per measurement well. |
| `graphics` | A character specifying the plotting method. For `"mean"`, a dose-response curve of the mean values of the measurement series is given, whereas one curve is plotted for each if `"single"` is specified. For `"fitted"`, a sigmoid-shaped derivation of the logistic model is fitted to the data. |
| `outdir` | The directory where the results will be written. |

### Details

In cytotoxicity screens of chemical compounds, biological activity is
typically indicated by the concentration for which a particular
proportion (typically 0.5) of cell growth is inhibited after a predefined
treatment period. For this purpose, all concentrations are plotted
against the percentages of cells still being alive under this
treatment, forming a dose-response curve under which the preimage of the 0.5
point is defined as the half-maximum inhibitory concentration
(IC50). For high-throughput screens (HTS), in particular, the
evaluation of the data needs to be performed in an automatic fashion.

The data input for the script is performed by tab-delimited data files
which are the typical output from appropriate microplate readers. A
character vector of file names is therefore expected as the first
argument to the functions. If 96- or 384-well plates are used for the
screen, the arrangement of the wells is in principle arbitrary. The
design must be specified by three tab-delimited files with one for the
coordinates of the measurement wells, one for the control wells and
one for the concentrations of the respective compound. Several
examples of each of these files are given in the `inst` folder,
e.g. the files `"default384_measure.txt"`,
`"default384_control.txt"` and
`"default384_dilution.txt"`. Details on the arrangement of these
files are given in the documentation of the corresponding data sets,
e.g. for `default384_measure`. In addition, a tutorial document
describing how to prepare the data and configuration is included in
the `ic50` package.

For each compound in the screen, a graphics output is given in the
file `"dose_response_curves.pdf"` in the output directory, where the screen
data are displayed as specified by the argument `graphics`. In
addition, quantitative results are written to a file `"ic50.txt"`
in the same directory. Inhibitory concentrations are calculated for
each of the curves and are given together with the respective
confidence intervals. The measurement accuracy is evaluated by the
maximum of the standard deviations at the respective concentrations
and by the coefficient of variation of the concentration values as
determined from the single replicates. Finally, the normalized data
rows detected from the plates in use are written to the file
`"measurement.txt"`, combined in one group for each compound.

### Value

A data frame with the following variables:

|  |  |
| --- | --- |
| `compound` | Compound names. |
| `ic50` | The inhibitory concentrations for the respective compounds. |
| `clow` | Lower 0.95 confidence limits for the IC values. |
| `cup` | Upper 0.95 confidence limits for the IC values. |
| `maxsd` | Maximum of the standard deviations at the measured concentrations as determined from the single replicates. |
| `cv` | Coefficient of variation of the IC values as determined from the single replicates. |

### Note

The nonlinear regression for the sigmoidal-shaped curve is **not**
performed by the least-squares method. Instead, the parameters are
adapted to the data by assumptions on the shape of an "ideal" curve such
as location and bending.

### Author(s)

Peter Frommolt, University of Cologne peter.frommolt@uni-koeln.de  
http://www.medizin.uni-koeln.de/kai/imsie/homepages/Peter.Frommolt/

### Examples

```
#Example from a cell line screen (2007). IC50 values are determined for
#the lung cancer cell line HCC2429 and 7 selected compounds.

data(HCC2429_1,HCC2429_2)
write.table(HCC2429_1,file="HCC2429_1.txt",row.names=FALSE,col.names=FALSE,sep="\t")
write.table(HCC2429_2,file="HCC2429_2.txt",row.names=FALSE,col.names=FALSE,sep="\t")

data(mpi384_measure,mpi384_control,mpi384_dilution)
write.table(mpi384_measure,file="mpi384_measure.txt",row.names=FALSE,col.names=FALSE,sep="\t")
write.table(mpi384_control,file="mpi384_control.txt",row.names=FALSE,col.names=FALSE,sep="\t")
write.table(mpi384_dilution,file="mpi384_dilution.txt",row.names=FALSE,col.names=FALSE,sep="\t")

print(ic50.384(files=c("HCC2429_1.txt","HCC2429_2.txt"),
               measure="mpi384_measure.txt",control="mpi384_control.txt",dilution="mpi384_dilution.txt",
               inhib=rep(0.5,7),outdir="./HCC2429_results",normalize="mean"))
```

---

[Package *ic50* version 1.3 Index]
