# Supplementary material for: Standardized high-throughput evaluation of cell-based compound screens
Source: BMC Bioinformatics. 2008 Nov 12;9:475. doi: 10.1186/1471-2105-9-475 (PMC2639430; doi:10.1186/1471-2105-9-475)
Supplement: Additional file 4 — Windows binary code of the software. A pre-compiled version is provided for MS Windows. It can be installed from within the R environment on Windows systems. [file 1471-2105-9-475-S4.zip › ic50/html/nsclc.html]

R: Results from a compound screen on 84 NSCLC cell lines.

|  |  |
| --- | --- |
| nsclc {ic50} | R Documentation |

## Results from a compound screen on 84 NSCLC cell lines.

### Description

For purpose of computational, lesion-based prediction of compound
activity in 84 non-small cell lung cancer (NSCLC) cell lines, these
were treated with a selection of compounds to measure the respective
concentration where 50 per cent of cell growth was inhibited after a
predefined time period. Most of the screening experiments were
performed on 384 (16 x 24)-well plates, were the experimental setup was
designed according to the arrangement in the files
`"mpi384_measure.txt"`, `"mpi384_control.txt"` and
`"mpi384_dilution.txt"` that are installed together with the
package.

These data sets are the direct output from a signal reader of the
384-well plates for the A549, Calu1, H322 and H2429 cancer cell lines
selected from the 84 NSCLC cell lines collection. A tab-delimited version
is installed together with the package.

---

[Package *ic50* version 1.3 Index]
